# Legitimacy of Front-of-Pack Nutrition Labels: Controversy Over the Deployment of the Nutri-Score in Italy

**DOI:** 10.34172/ijhpm.2022.6127

**Published:** 2022-02-20

**Authors:** Morgane Fialon, Lydiane Nabec, Chantal Julia

**Affiliations:** ^1^Nutritional Epidemiology Research Team (EREN), Sorbonne Paris Nord University/INSERM U1153/INRAE U1125/CNAM, Epidemiology and Statistics Research Center, University of Paris (CRESS), Paris, France.; ^2^Centre de Recherche Réseaux, Innovation, Territoire et Mondialisation (RITM), Université Paris-Saclay, Paris, France.; ^3^Public Health Department, Avicenne Hospital, Assistance Publique des Hôpitaux de Paris (AP-HP), Paris, France.

**Keywords:** Front-of-Pack, Nutri-Score, Stakeholder Theory, Italy, Legitimacy

## Abstract

**Background:** Front-of-pack nutrition labels (FoPLs) aim at increasing transparency and consumers’ awareness of the nutritional composition of pre-packed food products in order to improve the nutritional quality of their food choices. Nevertheless, the legitimacy of the Nutri-Score - the FoPL officially adopted in France and several other European countries - is subject to both technical and political controversy, particularly in Italy. In this study, we investigated how and by whom the legitimacy of the Nutri-Score, recognized by several institutional authorities, could be deconstructed within a specific system of norms, values and beliefs among Italian stakeholders.

**Methods:** A netnography completed with qualitative interviews with eight Italian and French nutrition and public health experts were carried out to highlight the dimensions (pragmatic, normative and cognitive) in which the Nutri-Score’s legitimacy is being challenged among the stakeholders involved in FoPLs’ implementation in Italy. The degree of influence and the position of these stakeholders on the debate around the Nutri-Score were assessed through the Stakeholder Theory (SHT), using their respective level of power, legitimacy and urgency. Furthermore, we compared the Italian and the French contexts on the issue.

**Results:** The direct implication of political parties and media outlets in framing the Italian debate around Nutri-Score as well as the high influence of corporate unions, led to a different political outcome than in France. Results also show that the deconstruction of the legitimacy of the Nutri-Score in Italy pertained mainly to its pragmatic dimension according to the Italian public health experts. Nevertheless, its two other dimensions (normative and cognitive) are also questioned by high-influence stakeholders.

**Conclusion:** Due to the limited mobilization of scientific expertise over the issue, the debate in Italy stayed centered around the "attack" of the Nutri-Score to the Italian way of life, mixing up concepts such as Made in Italy products and the Mediterranean diet.

## Introduction

**Key Messages**

** Implications for policy makers**

- The legitimacy of Nutri-Score label is being challenged by Italian experts, mainly on its pragmatic legitimacy (format per 100 g, colors, algorithm).
- For Italian stakeholders, a front-of-pack nutrition label (FoPL) is seen as incompatible with traditional products that cannot be reformulated and that are part of the Italian heritage.
- The higher proportion of speeches by trade unions and political parties on the Nutri-Score in the Italian media outlets compared to those by Italian public health experts led to a questioning of the cognitive legitimacy of the Nutri-Score (inaccurate reporting and statements).
- Stakeholders’ analyses could be conducted in order to ensure constructive debates in future contexts of FoPLs’ implementation and prevent inaccurate reporting and economically centred debates.

** Implications for the public**

Front-of-pack nutrition labels (FoPLs) are considered effective means of achieving health benefits in populations by orienting consumers food choices toward healthier options and encouraging reformulation towards a healthier food environment. Contextual debates over FoPLs involve multiple stakeholders whose influence may lead to policy decisions that could in turn impact public health. Greater understanding is needed on how stakeholders are involved in the implementation of a FoPL in a country, their influence, and the context specificities which can affect the perceived legitimacy of a FoPL and its potential implementation. Our study highlighted the limited mobilization of scientific expertise in particular in public health over the debate on Nutri-Score in Italy showing the challenges associated with the implementation of policies in nutrition which impact multiple sectors, and the importance of the framing of the debate on its outcome.

National and international public health expert committees have advocated for the implementation of interpretive front-of-pack nutrition labels (FoPLs) as an effective policy to encourage consumers to adopt healthier eating habits.^1,2^ In the European Union (EU), the Commission is expected to adopt a single and mandatory FoPL by the end of 2022 as announced in the Farm to Fork strategy,^3^ yet multiple schemes currently co-exist, including nutrient-specific formats (eg, the Reference Intakes implemented by several food manufacturers^4^), endorsement schemes (eg, the Green Keyhole in Scandinavian countries^5^) and summary graded indicators such as Nutri-Score, displayed on pre-packed food products in France since 2017 and adopted in several European countries on a voluntary basis.^6^ A report from the Joint Research Center (the European Commission’s science and knowledge service) providing insights on each of these labels according to evidence concluded that interpretive FoPLs using color coding were associated with better understanding and could guide consumers towards more health conscious food choices.^7^ Nutri-Score is a summary, graded, color-coded FoPL (with five categories from dark green/A to dark orange/E) with a two-fold objective: (1) to help consumers identify the overall nutritional quality of food products and guide them towards healthier food choices, and (2) to encourage manufacturers to reformulate the nutritional composition of food products. The Nutri-Score was introduced in France on the basis of numerous independent scientific studies investigating its performance against other types of labeling systems.^8^ The large multi-country FOP-ICE study in 12 European countries, including Italy in 2020,^9,10^ showed that the Nutri-Score appeared as the best scheme to help participants identify healthier food products compared to other FoPLs.

In the context of a gradual adoption of the Nutri-Score by multiple international stakeholders and EU member states, Italy engaged in a form of state lobbying against its development. The Ministry of Agricultural, Food and Forestry Policies (MiPAAF) aligned itself with the position of the national food and agriculture trade associations, suggesting that Nutri-Score would wrongly penalize products from the Mediterranean diet as well as traditional Italian products.^11,12^ In a press release of May 6, 2019, the representative of the Italian government for the World Health Organization (WHO), Ambassador Gian Lorenzo Cornado, stated that the concept of “nutrient profiles” of foods underlying FoPLs was “an entirely political concept with no scientific foundation.”^13^ Following this controversy, an alternative FoPL was developed in Italy in reaction to the Nutri-Score, the NutrInform Battery, an informative multi-nutrient label indicating the nutrient content provided for a portion of product consumed, adopted officially in October 2020.^14^ Of note, the Italian NutrInform Batterynever appeared as the subject of debate itself, and has not been implemented by stakeholders on the market to date. In September 2020, at the Agriculture and Fisheries Council of the EU, Italy with six other countries (Czech Republic, Cyprus, Greece, Latvia, Romania and Hungary) presented a document contesting some key components of the Nutri-Score as a FoPL, questioning in particular the use of color coding and a format per 100 g.^15,16^

 Given the debate surrounding nutrition labeling to improve consumer food consumption, this research aims at understanding who are the actors involved in the implementation of a FoPL in Italy and how their levels of influence, as well as their interactions, lead to a questioning of the legitimacy of the Nutri-Score.

###  Theoretical Background

The Stakeholder Theory (SHT) developed by Mitchell, Agle and Wood in 1997 provides a relevant framework to investigate the stakeholders involved in the development and implementation of a FoPL in Italy. The SHT is a set of theories which concept was explained by Roux et al: “Originally formulated in 1968 by Ansoff, the SHT introduces the idea that companies must consider the divergent interests of groups whose collective behavior may directly affect the future of the organization without being under its direct control, or who are affected by the company’s policies and practices and consider themselves to have an interest in the business.”^17,18^ The SHT has been introduced into the public domain and has been applied to managerial decision-making in a governmental context,^19^ in the case of consumer organizations,^20^ or to investigate the Hungarian Alcohol policy.^21^ According to Mitchell et al,^22^ stakeholders are evaluated using three criteria: power, legitimacy and urgency. Power is defined as: “the ability of a stakeholder to act to obtain the decisions they want”; legitimacy is considered as: “the general perception that the actions of the stakeholder are desirable, adequate or appropriate within a system of beliefs, values and social norms.” Finally, urgency captures “the critical nature of the stakeholder’s claims and the immediacy with which the firm is required to respond to them.”^20^ Adding the attribute of urgency “helps move the model from static to dynamic.”^22^

SHT results were combined with the approach by Varvasovszky & Mckee,^21^ and Varvasovszky & Brugha^21,23^ to characterize the level of influence and the position of each stakeholder on FoPLs’ debate in Italy.

Beyond stakeholders,^24^ research shows that legitimacy can be applied to a number of objects, including FoPLs. In this case, legitimacy has been found to rest on three dimensions: the normative legitimacy of their stated objectives, the pragmatic legitimacy of their means of action and the cognitive legitimacy of their previous actions.^25,26^ In this context, one of the objectives of this research is to understand how the legitimacy of the Nutri-Score system, recognized in France and some European countries by institutional authorities and consumers associations,^27^ can be deconstructed within a specific system of norms, values and beliefs among Italian stakeholders.

 We conducted an analysis of the legitimacy of the Nutri-Score system in Italy among stakeholders involved in the implementation of a FoPL in the country, by understanding their level of influence according to their degree of power, legitimacy and urgency to act for (/against) it.

## Materials and Methods

###  Data Collection

**Figure 1:**
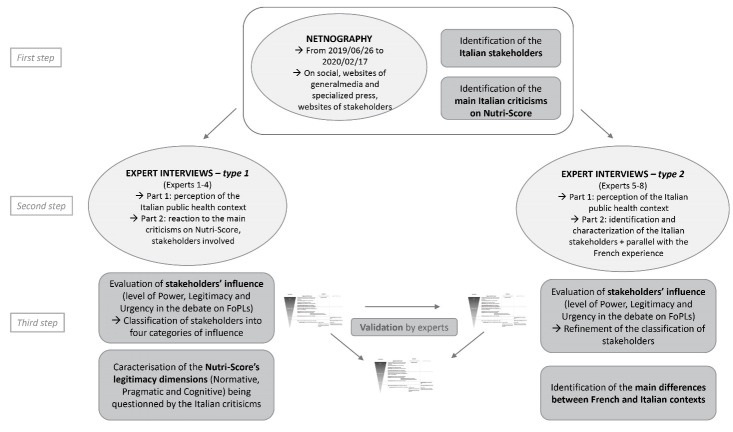
Methodology Steps and Their Applications. Abbreviation: FoPLs, Front-of-pack nutrition labels.

This empirical research was conducted using complementary approaches (Figure 1).

####  First Step

We carried out a netnography over several months to identify the main stakeholders involved in the debate over the implementation of a FoPL in Italy. The arguments for or against Nutri-Score were collected. This netnography included the social media platform *Twitter*, stakeholders’ websites and online articles from the Italian general and specialized press from June 26, 2019 to February 17, 2020. The dates elected for the netnography corresponded to a period where the debate over FoPLs and Nutri-Score peaked in Italy.^28^ Between June 26, 2019 and February 17, 2020, 247 online articles were found via Google News using the keywords “Nutri-Score Italia” and 93 were actually about Nutri-Score.

####  Second Step 

We conducted semi-directive interviews with eight experts in the field of public health and/or nutrition directly involved in FoPLs development and/or implementation whose opinions were not frequently relayed in the Italian media although they had high legitimacy on the issue. These interviews allowed us to gain an in-depth understanding of the Italian situation and to identify the stakeholders’ positions on Nutri-Score’s implementation that had not been found through the netnography as well as new stakeholders. To perform comparisons and understand the differences of contexts between Italy and France, we interviewed experts involved in the French debate on Nutri-Score and its international development. In total, six Italian experts from media, consumer associations, public health institutions and one with background in Italian trade associations were interviewed (Experts 1-4 and 7-8) as well as two French experts from the French directorate of health and a French research structure (Experts 5-6) (see Supplementary file 1).

We conducted two types of interviews (Figure 1). With the first four Italian experts (Expert 1-4) we explored the Italian public health context (general health status of the population and national policies in nutrition); we asked them to identify and to characterize the main stakeholders and to react to the main arguments collected in the netnography. In the next four interviews (Experts 5-8), we focused on the level of influence of the various stakeholders, and we invited French experts (and one Italian Expert working in France – Expert 5, 6, 7) to draw parallels between the Italian and the French experiences. All interviews were conducted and recorded in French, Italian or English and then fully transcribed and translated.

## Methods

As a third step (Figure 1), we performed a content analysis of the interviews to evaluate stakeholders’ influence through the characterization of their level power, legitimacy and urgency following the methodology proposed by Roux et al,^20^ derived from the theory proposed by Mitchell et al.^22^ The content analysis classified verbatim records from the different interviews according to the designated stakeholder. The level of influence and positions of key stakeholders emerged from first and second steps. A map placing stakeholders according to their power, legitimacy and urgency (as dichotomous attributes) was developed and used with Experts to validate the respective position of each stakeholder. Then, the attributes were combined to define their level of influence,^23^ whereby each additional attribute increased their level of influence, with the ‘power’ attribute being necessary to be qualified with ‘high’ influence. This resulted in four categories of stakeholders from high to low influence. We also defined three categories of position towards the Nutri-Score: Support, Opposition and Non-mobilized/Neutral.^21^

For the analysis of the legitimacy of the Nutri-Score in Italy, we relied on Suchman’s definition of legitimacy^24^ to analyze the arguments raised against the Nutri-Score according to its three dimensions (normative, pragmatic and cognitive). Elements of discourse from each interview or from content from the netnography belonging to one or several dimensions of legitimacy were reported and classified.

## Results

**Figure 2:**
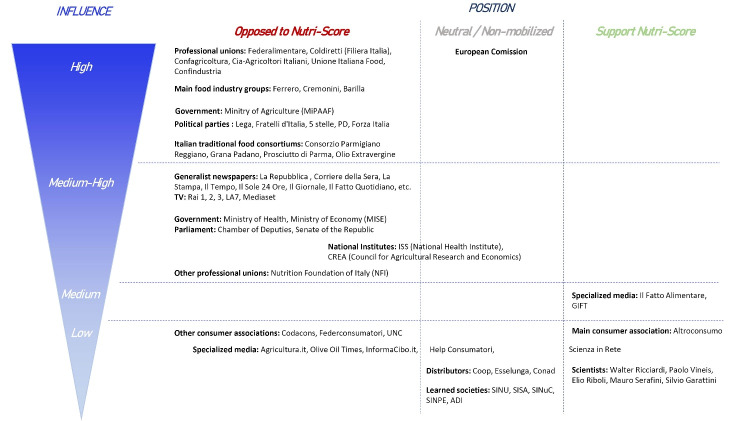
Stakeholders’ Influence and Position on Nutri-Score’s Debate in Italy. Abbreviations: MiPAAF, Ministry of Agricultural, Food and Forestry Policies; GIFT, Great Italian Food Trade; PD, Democratic Party; MISE, Ministry of Economic Development; GIFT, Great Italian Food Trade; UNC, National Consumers Union; SINU, Italian Society of Human Nutrition; SISA, Italian Society of Food Science and Nutrition; SINuC, Italian Society of Clinical Nutrition and Metabolism; SINPE, Italian Society of Artificial Nutrition and Metabolism; ADI, Italian Association of Dietetics and Clinical Nutrition.

If Italy and France could be seen as rather similar contexts in terms of food culture or political and economic contexts, we found differences in stakeholders’ involvement in Italy compared to the French context when adopting Nutri-Score. We represented on Figure 2 the Italian stakeholders taking part in the debate on Nutri-Score according to their level of influence and position (Figure 2). Statements from the netnography in support of the characterization of stakeholders are presented in Supplementary file 2.

###  Stakeholders Influence and Position on Nutri-Score’s Debate in Italy, Main Differences With the French Context

####  High Influence Stakeholders: A Consensus of Trade Unions and Political Parties Over the Opposition to Nutri-Score

Recurrent actors were identified through the netnography, and their frequent presence in the media was a first demonstration of their high level of power in the Italian system. After Nestlé’s Public announcement to deploy Nutri-Score on all its products sold in Europe (only in countries where Nutri-Score was authorized) on June 26, 2019,^29^ immediate reactions of Italian corporate unions appeared in the press. We identified two main categories of trade unions involved in the debate on FoPL with Confindustria being the biggest entity representing Italian companies. There were, on one side, the representatives of the agricultural sector with Coldiretti, Confagricoltura and Cia-Agricoltori Italiani; and on the other side, the representatives of the food industry sector with Unione Italiana Food and Federalimentare. Coldiretti and Federalimentare were the most represented organizations in the media and their discourses were based on three main arguments: Nutri-Score wrongly penalizes *Made in Italy* and Mediterranean diet’s products which represent healthy and natural foods that are traditional; the whole diet of an individual has to be considered instead of reasoning on the individual foods consumed; and finally FoPLs should contribute to the education of the consumer in reading the nutrition declaration (Supplementary file 2). Then, other unions of producers also reacted such as Cia-Agricoltori Italiani using as examples Italian traditional products that were found in the majority of stakeholders’ statements (Parmigiano Reggiano, Grana Padano, Parma ham, olive oil). La Repubblica, a major newspaper in Italy released an article^30^ a few days after Nestlé’s announcement, citing the arguments of Coldiretti, Federalimentare, Cia Agricoltori Italiani as well as Filiera Italia, a recent association created in 2017 by Coldiretti that is “dedicated to the enhancement and promotion of *Made in Italy* agri-food excellence.”^31^ The French Experts we interviewed highlighted some similarities with the context in France at the time of the implementation of Nutri-Score: *“This is something that we have even experienced in France, when you look at the discourse at the beginning of the National Association of Food Industries (ANIA), it was always supposedly to defend the small companies. Saying that they are going to be victims of the system, but it was the big groups like Nestlé, Mars or Kellogg’s, who made their corporation use this argument of defense of the small producers. We can see that in Italy, those who express themselves are big structures like Coldiretti, which makes an extremely strong lobbying, but each time trying to put forward two arguments that work very well in Italy: the defense of small producers and on the other hand, the infringement of the strong values of the Italian culture” *(Expert 5). The parallel with the French debate was also mentioned by Expert 6: *“The speech you must have read was for a long time the one we had in France, also from the ANIA. So, officially, in the positions that have been taken by the Italians, it is, we do not want this discrimination between foods because we are Italians, we have our food culture and we have in this food culture a lot of artisanal products or quality products on other criteria than nutritional quality which will be in particular Parma ham, Parmigiano Reggiano...”*

Represented through these manufacturer associations, Italian agri-food sector includes major national food companies as Cremonini, Barilla and Ferrero in terms of turnovers^32^ and food consortiums such as the Consorzio Parmigiano Reggiano. Apart from the reactions of Consorzio Parmigiano Reggiano and Consorzio Grana Padano that expressed their opposition to all kinds of FoPLs on their products – including NutrInform battery – direct reactions from national food companies and other food consortiums were quasi-absent from the media. Nevertheless, the experts highlighted that these entities were also opposed to Nutri-Score and had a high influence. Expert 8 identified Ferrero as a key stakeholder with strong power in the debate on FoPL in Italy: “*Nowadays Ferrero has a crucial role in Federalimentare decision making process and it has the full control of Italian politics in agriculture even through Coldiretti, and the marriage between Ferrero and Coldiretti against nutrient profiling systems and against Nutri-Score was made clear some years ago when a document was published by Nomisma,*^33,34^* financed by Ferrero, and published by Federalimentare and Coldiretti in Brussels with the objective of opposing nutrient profiling.”* Several experts mentioned the influence of corporate unions and the food industry on Italian decision-makers: *“All the Italian governments of the right, left, center are opposed to the Nutri-Score, to the traffic lights of the UK because in Italy the food lobby is very strong, and everything that is against Italian products is not supported” *(Expert 1) and on political parties such as the Lega:* “A few days before [Matteo Salvini’s first declaration on Nutri-Score], producers’ representatives had issued a press release that was very, very negative against Nutri-Score and supported an alternative label [ NutrInform Battery]. And so Matteo Salvini relied on that. And he quoted exactly the numbers that these people had said. He cited exactly the examples that these people had said, and as a result, the debate was really, from the beginning, managed by these actors, Federalimentare etc, they were the ones who laid the foundations of the debate” *(Expert 7).

The implication of the Lega, a populist Italian political party, started in December 2019 in the context of Matteo Salvini’s campaign for 2020 regional elections in Lombardy. Matteo Salvini, its Federal Secretary, brought the topic of Nutri-Score through his Twitter account and several TV talk shows such as “Porta a Porta.” Using the same arguments as the agricultural corporate unions, he positioned Nutri-Score as a threat for the Mediterranean diet and *Made in Italy* products and even as a scheme from Europe, framing it outside public health stakes.His opinions were also largely relayed by the Italian press (Supplementary file 2) which provoked a new momentum to the Nutri-Score debate in Italy; Expert 6 insisted on M. Salvini influence in this debate: *“In a political context that was complicated in Italy he [M. Salvini ] framed the debate in such a way that no other party could go against this vision: We Italians have good food. And it’s true, they have good food and if I oppose the Nutri-Score by showing that it is not adapted to Italian food and that behind it there is Europe, I create an anti-European political condition etc, therefore completely beyond the framework of health” *(Expert 6).The opposition of many other Italian political parties from the right and left were also relayed by the press (Supplementary file 2) highlighting the agreement of the political actors in Italy on the topic of Nutri-Score. In France, as Nutri-Score was presented in the frame of health only, politicization of the debate did not emerge, Expert 6 highlighted this difference with the Italian context: *“I think that we have reached a very high level of politicization of the Nutri-Score in Italy where in France we did not have it in this way because as we often see in the field of nutrition, I experienced it for a period of about 20 years, in France, the nutritional policy, we do not classify it on the right or on the left, there are always in the Parliament, deputies of different sides who will support positions on public health against economic positions”* (Expert 6).

The way the Italian government reacted to the situation was also quite different than in France. It showed its opposition by supporting the creation of an alternative FoPL, the NutrInform Battery, that was presented as an initiative of four ministries^35^ whereas in France, Nutri-Score was first presented as an initiative of the Ministry of Health with the inclusion of other Ministries at a later stage.^6^ Also, the role of the FoPL was presented differently in the two countries. Italy positioned notably its FoPL as a solution to *“protect Made in Italy products”*^35^ mainly through speeches by the Ministry of Agricultural Policies whereas in France, Nutri-Score was presented as a public health tool to make it easier for consumers to understand nutritional information and thus help them make informed choices.^6^

####  Medium High Influence Stakeholders: The Role of the Main Media Outlets in Framing the Italian Debate With Little Space Given to Stakeholders With High Legitimacy on Public Health Issues

Interventions of the Health Ministry in the media were less frequent compared the Ministry of Agriculture on the debate around Nutri-Score; Expert 1 highlighted the fact that there were few health information campaigns on nutrition: *“There are really few public health messages, the Ministry of Health sometimes does campaigns but they are always about vaccines, women’s health,... but not much about food.”* Some issues in the way public health nutrition messages are communicated to the public was also highlighted: “*The Healthy Eating Guidelines is a political document. The final user is the consumer, but they are written in a language that not all consumers are able to understand. […] There are 229 pages. It is unthinkable that an average consumer would read 229 pages”* (Expert 3). The Italian and the French Health Ministries appear to have different level of influence on topics related to food and nutrition, as Expert 6 told us: *“In France, if you want, I think that on subjects like that, in general, health is very, very good at being the white knight who is going to attack the bad industrialists because globally, the idea in the population in France, it is still that, the ‘bad’ industrialists, that’s our political culture.”*

As reported in Supplementary file 2, the Italian Parliament’s vote against Nutri-Score was relayed by the press and mentioned by some experts: *“The vote of the decree condemning Nutri-Score and proposing an alternative was signed unanimously by the Parliament” *(Expert 5).

The National Health Institute (ISS) and the Council for Agricultural Research and Economics, which are the public health institutes under the Ministry of Health and the Ministry of Agriculture (MiPAAF) respectively, have both participated in the development of the Italian FoPL NutrInform Battery but we did not identify any occurrence stating their position on Nutri-Score in the netnography. Their legitimacy on the topic was highlighted by Expert 7: *“Yes I know them [...] if legitimacy is institutional legitimacy, then clearly, they have it.”*

The role of the main media outlets also differed compared to France where investigative newspapers and TV shows seized the subject of Nutri-Score and pointed out the lobbying of the food and the agriculture industry. In Italy, prominent media outlets appeared to repeat arguments from the corporate unions or political parties without questioning them. Media’s influence on the debate was reinforced with the rapidity with which such complex topics as the implementation of FoPLs were treated with very little space for stakeholders with high legitimacy in terms of public health. Expert 7 highlighted the importance of television at the stage of political debates: “*Matteo Salvini launched the debate [on Nutri-Score], I think at the end of January or something like that, and it was on Rai 1, which is the most important TV channel. And he did it in an evening talk show called Porta a Porta, it is the most important talk show of Italian politics, it is where politics is made.”* Expert 7 also mentioned that these TV shows talked about Nutri-Score always in the same way and only from one viewpoint: *“I think Italians watch TV a lot, more than newspapers for example. I know that on the TV shows, they had made subjects on the Nutri-Score, almost all the channels. It was unanimous, that is to say that the approach was always the same. It was always to say the Nutri-Score is zero, it’s against our products, etc. So, it was the classic image where there was a basket with beautiful Italian products and then the presenter would say, ‘Those French people don’t want us to eat these products etc.’ It was very characterized, in a nationalist way, and it was very, very strongly leaning to one side....”* Indeed, as it was noticed by Expert 5 and through the netnography, the arguments put forward by the trade unions were not questioned by media investigations as it was the case in France with TV shows such as Cash Investigation: “*Obviously, there may not be a Cash Investigation there, but I spoke with journalists from Le Monde, Médiapart, Le Canard enchaîné in France, they say that there are investigative journalists in Italy but on this theme nobody has taken it up. So, what are the reasons behind it?”*

The Nutrition Foundation of Italy (NFI), an organization grouping committee of experts and industrialists was also mentioned as a stakeholder taking part in the debate and opposed to Nutri-Score: *“I would say there’s more balance between multinationals like Nestlé, Unilever, Danone and national champions like Ferrero and Barilla [inside NFI] but they know how the power is distributed in Italy. So, when Ferrero, Barilla, Unione Italia Food order to write a position against [Nutri-Score] they do it” *(Expert 8).

####  Medium to Low Influence Stakeholders: Some Italian Stakeholders in Favor of Nutri-Score and the Differences With France on the Implication of Consumer Associations and Retailers

The few actors supporting Nutri-Score had high legitimacy on the topic but their low power resulted in an overall medium to low influence that was insufficient to shift the debate. This was the case of specialized media such as Il Fatto Alimentare or GIFT (Great Italian Food Trade) that were the more active ones on the topic. They are independent Italian online newspapers specialized in topics related to food. Il Fatto Alimentare started covering the topic of Nutri-Score as soon as it was adopted in France back in 2017 and the topic of FoPLs in general even before. The netnography demonstrated their support in favor of Nutri-Score. Export 8 highlighted that: *“both Il Fatto Alimentare and GIFT have been pushing in favor of the Nutri-Score. Let say that Il Fatto Alimentare is a more popular website whereas GIFT is rather for professionals.”* The online journal Scienza in Rete that is specialized on scientific issues also took a stance by publishing a communiqué from a group of scientists in favor of Nutri-Score. Among scientists, Walter Ricciardi was identified a few times in the netnography, he appeared for instance in a TV show on the channel LA7^36^ but in general scientists had a low influence in the debate in Italy as expressed by Expert 4: *“Scientists can provide evidence, but decisions regarding the label are a matter for politics, for the decision-makers who make the final decision.”* Another part of specialized online media closer to the agricultural sector like Agricultura.it relayed the anti-Nutri-Score arguments of corporate unions (Supplementary file 2).

Regarding consumer associations, Italian ones didn’t have the same level of influence than in France where the main consumer association represented a key stakeholder in the debate on Nutri-Score. Altroconsumo, the main one in Italy which is part of the European Consumer Organization, expressed itself in favor of the Nutri-Score but its position was not widely reported in the media: “*We tried to involve Altroconsumo, which is the largest consumer association in Italy, but with little success. I don’t know what happened but I have the feeling that they tried to keep this subject quiet because they thought it was an unpopular topic in Italy and to be favorable could be dangerous for them so they didn’t really react” *(Expert 1). Other consumer associations like Condacons were rather opposed to Nutri-Score (Supplementary file 2).

Learned societies like SINU (Italian Society of Human Nutrition) did not communicate publicly on the subject until 2021, contrary to the French context: *“In France and in many other countries, learned societies have played an important role, not only the nutrition one, but also in public health, pediatrics, cardiology and cancerology” *(Expert 5).

With regards to retailers, while they would have some legitimacy to express themselves on this topic considering their stakes in food production and/or distribution, we did not find any occurrence of a public position on the debate from the netnography. Compared to the French context, this is also a major difference as explained by Expert 6: *“and if you want, I think that in France, in the way it happened, in the end the Nutri-Score won, beyond the scientific studies, from the moment Leclerc, Intermarché [French retailers] said we are going, for me, seen from the inside, it was an extremely strong element to make things change. So, in Italy, if you don’t have this firepower of the large-scale retailer on consumers, I think it’s much more complicated and that, as a result, the industry can have more impact.”*

###  Main Criticisms of the Nutri-Score System and the Questioning of its Legitimacy by Italian Experts

 The netnography allowed data collection on the main criticisms on Nutri-Score in the discourses of Italian stakeholders. Four main criticisms were used in the experts’ interviews to collect their reactions and opinions as presented in Table. Experts 1-4 were interviewed on this topic.

**Table:** Experts’ Reactions to the Four Main Criticisms on Nutri-Score in Italy

**Main Criticisms Highlighted by the Netnography**	**Underlying Topics Highlighted by Experts**	**Expert Opinion**	**Expert**
Nutri-Score penalizes the Mediterranean diet/*Made in Italy* products	Lobbying around Mediterranean diet	*“I think that in Italy, there is this convention that Italian products are Mediterranean diet’s foods and politicians, lobbies, exploit the ignorance of the population. The Italians do not really know what the Mediterranean diet is and they exploit it to make the population believe that all Italian products are part of the Mediterranean diet and they exploit these narratives to attack the Nutri-Score. So, if all Italian products are the Mediterranean diet, a label that penalizes an Italian product attacks the Mediterranean diet and this is a reason that is really effective among the population. Whereas the Nutri-Score can penalize in the same way a French, German, etc product that has the same characteristics.”*	Expert 1
	Traditional food products cannot be reformulated	*“One of the aims of the Nutri-Score system is pushing the producer to the reformulation of the Red labelled products. And, you can bring to reformulation the products with very heavy industrial transformation or industrially made, for example, snacks. But in Italy, several products of the Mediterranean diet have their recipe which was brought by a very long tradition, a very long historical background. And they have, do you know ‘ disciplinare,’ for example, the recipe of Parmigiano Reggiano is set by law, we have a law that say... And so also, Parmigiano Reggiano is made after a very long period into the canteen. Okay. So, you cannot bring to the reformulation a product with a natural, with a natural transformation such as the ham or such as the Parmigiano Reggiano.”*	Expert 4
Nutri-Score is not scientifically based	Inaccurate reporting	*“Today the color for the olive oil has been changed for yellow but they [articles’ readers] continue to say red light for the olive oil. Even if I answer, no the olive oil today has the yellow light because the French recommendations as the Italian ones say to consume extra virgin olive oil but then again, the following comments: ‘the olive oil has the red light.’ I think the public conversation around the Nutri-Score has been polluted by these false narratives, fake news, [from politics and trade unions] it's really hard to have a conversation based on facts.”*	Expert 1
	Other evidences needed	*“Rather Nutri-Score system has a solid scientific basis, but in my opinion, it lacks the most important thing, the one that would interest me and that is: the Nutri-Score reduces obesity and that, I don't know.”*	Expert 3
		*“Nutri-Score has an algorithm, you know, it considers some healthy nutrients and unhealthy nutrients. Maybe I do not agree about the weight of every nutrients in the algorithm. For example, I don't like that proteins are considered as healthy nutrients. But for example, the body of evidence supporting the impact of the Nutri-Score on the market, on the consumer choice, is very good.” *	Expert 4
The Nutri-Score’s algorithm and/or its format are not adapted	Format per 100 g or per portion	*“The portion is the most important thing, it is necessary to make the citizen understand that the number of calories, the amount of nutrients is contained in the portion, this is the point where we should insist. Otherwise we can have something that is not easy to understand for consumers. For example, if we take the pizza Margherita, with the Nutri-Score we can give it the color green for 100 g of pizza, but we will eat 300 g of pizza, so this color is not valid. It's the same for vegetable oils, rapeseed, sunflower, olive, etc which have a red value but in reality, we're going to eat a very small amount.”*	Expert 2
		*“This is another big difference between Nutri-Score and NutrInform and it is also an issue that brings a lot of discussion. And of course, 100 g is universal, in any country around the world, 100 g is 100 g, ok, and this is very good to compare food products. On the other hand, you don't eat 100 grams of a lot of food, for example, olive oil. So as long as you have to compare two different foods in the same categories, 100 g, the indication of the 100 g works very well. But, when you have to build your diet, in my opinion, this is based on my belief or my scientific belief but of course this an opinion, when you have to build your personal diet, and this is the aim of the NutrInform Battery, you cannot use 100 g because, for example, the portion of olive oil is 5 g. On the other end, the portion of a deep-frozen pizza is 250 g. So, I think that 100 g in building your diet can bring some problems. On the other hand, with NutrInform we have to fix the portion. NutrInform can work well in this regard only if we have very fixed portions, because we cannot, for one mozzarella, use the portion of 80 g and for another mozzarella use the portion of 120 g, because otherwise, the consumer gets very confused. So, I think that Nutri-Score uses 100 g and NutrInform uses the portion because the aim of those two FoPLs are different. They are thought to work differently on the market.*”	Expert 4
	Misunderstanding of the format with colors	*“In Italy there is strong opposition to red, food producers do not want the color red on their products and there are also many consumers who think that red will be interpreted as ‘not eating’ and not ‘eating in moderation’ as it should be. And that's why the NutrInform has no color, the problem is red and all the colors that are used on the Nutri-Score and English Traffic Lights. That's why even though we know that a label without colors is not efficient, they opted for labelling without colors because the problem is the colors and especially the red interpreted as a ‘stop, do not eat.’ But I think that if with the introduction of a label like the Nutri-Score there was a good institutional communication on how to interpret it, this problem could be avoided, but Italian institutions are not very good in official communication.”*	Expert 1
		*“I don't like a label that divides the food products in ‘good, healthy’ and ‘bad, unhealthy.’ I think that everything is about the frequency you eat... even if you eat every day only green labelled products, ‘A’ labelled products, you don't have a balanced diet. So, I don't like the red and the green because green is related to ‘OK, you can go as many times as you want’ and red to ‘stop, you can't.’ Unless you inform very, very well the population that the red doesn't mean ‘stop’ but just eat with moderation and the green means ‘okay, you can have it several times a day’, but you have to inform the population very, very well, because in my opinion, red is, in the general consideration, associated with ‘stop’ and green is ‘OK. You can go.’”*	Expert 4
	Proteins and saturated fats in the algorithm	*“And then one thing I don't understand, it's part of the lack of scientific evidence, is that I absolutely disagree with the fact that proteins can be a corrective factor. For me it is an aggravating factor to have proteins, it's worse, that is to say that in Italy we consume on average 1.4 g of proteins per kilo of body weight. However, if we consider that 1 kg of body weight is taken from a population half of which is overweight, we consume a disproportionate amount of proteins, so proteins should not be a correcting factor, fruits and vegetables should be, I agree.”*	Expert 3
		*“Saturated fatty acids are important, you have to eat them normally... And usually, I'm not going to eat a very high amount of butter for example, it's difficult to eat 100 g of butter...”*	Expert 2
Nutri-Score is oversimplifying	Education of the consumer	*“I don't like FoPLs in general, all of them, however, I think FoPL is the magic wand that the consumer wants. The consumer doesn't want a ‘yes but’, they want a ‘yes or no.’ The FoPL gives it. Today, some FoPLs give additional information. Other FoPLs are directional, ie, those with a color. I have several problems with this. The first problem is what I was saying, to move away from food education and have the consumer choose by looking at a color and not at the label, the back of the package thinking because it has this, because it has that, I will take that product.”*	Expert 3
		*“Education is the first step. We cannot achieve anything, any results without educating the population because the knowledge and the awareness of the people is the first step to reduce obesity. If you try to fight childhood obesity or adulthood obesity, in up to bottom strategies, it doesn't work. You have to work on the awareness and the knowledge or education of the population.”*	Expert 4
	Loss of the information per nutrient	*“I have met two groups of critics: those who think that the Nutri-Score is too simple, too reductive; and those who think that a label like the English one [Multiple Traffic Lights] is not working well because all nutrients are classified separately. And it's not easy to reconcile the two groups: those who think that the food should be considered as a whole like Nutri-Sore does; and those who think that each nutrient should be considered separately because for instance there are people who need to pay attention to salt because they have a cardiovascular problem, there are people who need to pay more attention to sugar, etc and it's not possible to satisfy everyone.”*	Expert 1
		*“So, this is the weakness, but maybe this is also the strength of the Nutri-Score. Because, when I go shopping, I don't have much time. I am in a hurry so I don't have time to read the GDA on the back. Unless I am very interested in one, one or more food categories, for example, I do this for cheeses. I'm trying to buy the cheese with the lowest fats, saturated fats, because I like cheese, but I can't have, for health reasons, too much saturated fats in my diet. So, for cheeses, I'm looking for the GDA on the back. But for the other food categories, I don't have time so I don't look at the nutritional facts, at the nutrient level. So, I think I agree that FoPL is a simplified information regarding the GDA on the back. Maybe, so, Nutri-Score simplifies this process for the consumers. So, if I don't know what to choose between two cheeses, I can choose the green one instead of the orange or the reddish one. But on the other side, I think that there is too many information packed into the color of the Nutri-Score because you have information about sugars, you have information about fats, you have information about salt so you don't know, with just the Nutri-Score, if that product is good because it has low salt or low fats or low sugars. So maybe Nutri-Score brings too much simplification.”*	Expert 4

Abbreviations: FoPLs. Front-of-pack nutrition labels; GDA, Guideline Daily Amounts.

The legitimacy of a FoPL relies on three dimensions^24,26^:

- The **normative legitimacy** of an organization is defined by: “the beliefs that its activity actually promotes social well-being as defined by its audience and its socially constructed value system.” In this case, this aspect means the relevance of Nutri-Score’s stated objectives for Italians.
- **Pragmatic legitimacy** is “based on the organization’s capacity to satisfy the interests of the various social actors.” In other words, are the means mobilized by the originator of the FoPL relevant, sufficient, neutral, according to Italians?
- **Cognitive legitimacy** is “based on the consistency between the organization’s behaviors and the patterns of what is understood by the social actors.” Meaning, the consumer awareness of the history, actions and existence of the Nutri-Score system.

**Pragmatic legitimacy** of the Nutri-Score seems to be the most criticized aspect by Italians actors. Many elements of the pragmatic legitimacy of the Nutri-Score have been questioned both in terms of its graphic format and its intrinsic algorithm. The Nutri-Score’s colorimetric scale from green for “A” to dark orange for “E” is seen as an indication that can be confusing for the consumer. The green color is suggested to induce people to eat the product in greater quantities by drawing a parallel with the effect on the consumption of “light” foods: *“The consumer considers ‘light’ food as something that he can eat as he pleases. And it’s the same thing, but we probably don’t have any studies that can say that, that what happens with green. Red worries me less because if you don’t eat it, that’s fine, green worries me more”* (Expert 3). The perceived risk is that the colors would classify foods as “good” or “bad” without considering the frequency of consumption, Expert 4: *“even if you eat only green labelled products every day, you are not eating a balanced diet. So, I don’t like red and green because green is related to ‘OK, you can go as many times as you want’ and red is related to ‘stop, you can’t.’”*; *“the colors will give confusing indications, they are not a correct indication, the correct indication is the quantity that we will eat and the contributions of these quantities to the daily consumption”* (Expert 2). These elements show that for the majority of Italian actors, Nutri-Score will not have the capacity to improve the diet of Italians and even could create some negative effects on consumer behaviors. Another criticism on Nutri-Score that confirms this belief is the loss of the information per nutrients. The reason why Nutri-Score is seen as an inappropriate FoPL is that it would jeopardize the education of the consumer, and in particular its education in reading and deciphering the nutritional declaration on food products (Expert 3, Table).

The various strategies on consumer’s education and the different visions on the role of a FoPL also questioned the **normative legitimacy** of Nutri-Score. Expert 4 pointed out one of these elements: *“So, I think that Nutri-Score uses 100g and NutrInform uses the portion because the aim of those two FoPLs are different. They are thought to work differently on the market” *(Table). According to Italian actors’ discourses, education of consumers and promotion of the Mediterranean diet are the main strategies to prevent nutrition-related diseases. The NutrInform Battery with its detailed graphic format is seen as a means to educate Italian consumers on nutrition. The NutrInform Battery would also be more appropriate for people with specific needs like diabetics for instance who have to track their consumption of sugars (diabetes prevalence is estimated at 5.3% in 2016 in Italy^37^ and 5.2% in 2019 in France^38^). Along with the education of consumers, promoting traditional foods from the Mediterranean diet is part of the Italian discourse as expressed by the Ministry of Agriculture: *“Consumers have the right to be correctly informed, and our food excellencies cannot be penalized by traffic lights [placed on food packaging], Bellanova added. ‘The promotion of a healthy diet requires a multidisciplinary approach. Nutrinform is our alternative to Nutri-Score, but it also is much better. It does not penalize [food], it does not say what is good or what is evil, it informs [the consumers]. [...] and it also defends the unique heritage of the Mediterranean diet.’”*^39^ Yet, the term Mediterranean diet may not be adequately interpreted in the population, (Expert 1, Table). Indeed, the most cited examples of traditional foods seen as penalized by Nutri-Score (Expert 4, Table), are Parmigiano Reggiano cheese, Prosciutto and olive oil. Except for olive oil, cheese and ham are to be consumed in small quantities in the Mediterranean diet.^40^

Finally, the **cognitive legitimacy** of Nutri-Score in Italy is also criticized. The netnography and the experts’ interviews showed a high circulation of inaccurate statements about Nutri-Score and no presentation of opposing viewpoints. Consequently, it is likely that Italian consumers have an incorrect perception of Nutri-Score resulting from this context. Expert 1 expressed his concern on this topic: *“So, I think that many Italians don’t know the true history of the Nutri-Score and have been convinced that it is an instrument of the industry, of the dominant powers of Europe against Italian food because part of the political and industrial world and lobbies have pushed these narratives.”* These aspects on the cognitive legitimacy of the Nutri-Score concur with the part “Nutri-Score is not scientifically based” in Table. All the scientific evidences surrounding the implementation of Nutri-Score have been kept quiet in the main Italian discourse although all of the experts interviewed do not agree with this statement and recognize the scientific background of Nutri-Score. Nevertheless, this argument is widely used by high-influence Italian actors and even among the government like the Ministry of Agriculture Teresa Bellanova when defending the Italian FoPL NutrInform Battery: *“ [...] the citizen who should not be misled by colors or images that have nothing scientific [...].” *^41^

## Discussion and Policy Implications

Overall, our analysis revealed that all Italian stakeholders with a high level of influence on the potential implementation of a FoPL in Italy and with the power to act upon it were opposed to Nutri-Score. The most mediatized aspects of their criticisms challenged the normative and cognitive legitimacy of Nutri-Score, highlighting different visions on FoPLs purposes and thereby refuting the scientific validation of Nutri-Score. Economic arguments based on *Made in Italy* products were dominant in trade unions and politicians’ discourses. Interviews with Italian experts from public health governmental institutions revealed that behind the mediatized side of the debate on Nutri-Score in Italy, the pragmatic legitimacy of Nutri-Score was at the basis of criticisms. Indeed, the disagreement with the use of colors, the format per 100 g or the algorithm of the Nutri-Score highlighted different visions of nutrition education between France and Italy. An interesting element is the similarity between the criticisms raised against the Nutri-Score in Italy during our study and in France at the inception of the debate in 2014. All the elements that have been identified in this study were also questioned during the debate in France (color-coding, use of portions vs. 100 g, traditional foods, etc), and even led to comparative studies of various FoPL formats, including one very similar to the NutrInform Battery system.^42,43^ Results from these comparative studies showed that Nutri-Score was the most efficient FoPL in conveying information on the nutritional quality of foods and thus helping consumers to discriminate between products, compared to the other proposed formats. Results pertaining to objective understanding in particular were later confirmed in the international FOP-ICE study – including a sample in Italy.^9^ Therefore, it appears that the Italian debate somewhat mirrors the French one, yet dismisses scientific results from the French experience. Nevertheless policy-makers should be encouraged to conduct comparative studies to ensure that they implement the most efficient scheme. In Portugal, for instance, a debate over the potential implementation of a FoPL – Nutri-Score also being one of the evaluated options – emerged and led to the mobilization of a collective scientific expertise and targeted studies.^44^ While the health impact assessment conducted in the Portuguese expertise did not conclude on which FoPL was the most adapted in the Portuguese context, it still gave new insights on FoPLs’ implementation in Portugal and left the debate opened for future studies. In Italy we were not able to identify a similar mobilization of scientific expertise, very few Italian scientists expressed themselves during the debate, and some were even put aside. The two Italian studies comparing Nutri-Score with other FoPLs were limited to the comparison with NutrInform Battery and explored only one of the dimensions of a FoPL (subjective understanding).^45,46^ These studies found that NutrInform Battery was perceived as an informative FoPL scheme by consumers and that it was more helpful than Nutri-Score in helping consumers understand the product’s nutrient composition.

Nutri-Score and NutrInform correspond to differing strategies toward improving consumers diet. In Italy, the strategy is focused on educating consumers about the Mediterranean diet and through the use of the NutrInform Battery which would allow them to measure and track their nutrient intakes during the day. However, this nutrient-based approach may appear somewhat in contradiction with the Mediterranean diet which is based on the promotion of certain food groups and not nutrients. Spain, where the Mediterranean diet is also a very strong component of dietary education and culture – though adherence is declining^47^ – has adopted Nutri-Score showing diverse strategies even among Mediterranean countries.

The prominence of the term “Mediterranean diet” could be noticed in the Italian discourse. Several papers have investigated adherence to the Mediterranean diet in Italy and highlighted a decreasing trend in adherence over time, in particular in younger generations and individuals in lower socio-economic groups.^48-50^ Along with this decrease in Mediterranean diet’s adherence, a report from WHO Childhood Obesity Surveillance Initiative^51^ showed that southern European countries including Italy had the highest rate of child obesity. These trends were also described by the experts interviewed. In terms of policy implications, two ways of addressing these issues could be devised. One strategy, defended by Italian stakeholders, is to take a step backward and “reintroduce” the Mediterranean diet among younger generations by focusing on nutrition education. The other strategy would be to take into account changing habits among new generations and the presence of lower education and socioeconomics groups (the overall level of inequalities has grown more in Italy than in several other developed countries over the last 25 years^52^) and provide nutritional tools that are adapted to these new ways of consumption and populations. Nutri-Score could be one of these tools as its graphical format with colors and letters makes it easily understandable^53,54^ for targeted populations. In a study that compared five FoPLs among 1032 Italian participants in terms of food choices and understanding of the labels by consumers, Nutri-Score demonstrated the highest overall performance in helping consumers to correctly rank the products according to their nutritional quality compared to the reference intakes.^55^ As a result, Nutri-Score could be seen as a tool for preventive action and awareness of nutritional issues along with a continuous education of the population.

Regarding strengths and limits of this study, the interviews conducted were based on an important phase of netnography that allowed the selection of experts involved in the debate from various stakeholder groups. While no expert from the industry or political parties were formally interviewed for this study, their points of view were widely disseminated in the media or through press releases and could be analyzed in our study. The application of the SHT to a public health policy and the analysis of the legitimacy of Nutri-Score were based on papers^20,26^ that used these methodologies in the case of consumer associations. One of the strengths of our study is therefore the innovative use of the SHT applied to a FoPL in order to analyze the impact of a specific context on the acceptability and the legitimacy of a public health policy such as the application of a new FoPL. One of the limitations however is that we used a simplified method for the validation of the classification of the Italian stakeholders by the experts, compared to Roux et al.^20^

## Conclusion

The analysis of the stakeholders involved in the deployment of a FoPL in Italy reveals major differences with the French context. The influence of public health structures compared to the food and the agriculture industry (which also represents traditional *Made in Italy* products) in Italy seems less pronounced, allowing the arguments of the latter to dominate the debate without their legitimacy beeing questioned in terms of public health expertise. This grey area also led to a politicization of the debate mixing up economic interests with public health motives. As a result, the debate in Italy stayed centered around the “attack” of the Nutri-Score to the Italian way of life, confusing concepts such as *Made in Italy* products and the Mediterranean diet. The limited mobilization of scientific expertise over the issue shows the challenges associated with the implementation of evidence-based public health policies.

## Acknowledgements

 The authors would like to thank all the Experts interviewed for their availability and their major contribution to this scientific paper.

## Ethical issues

 The experts were aware that the interviews would be recorded and the verbatims would be used in a scientific article. All data were anonymized. Under French law, no approval is required from an ethics committee.

## Competing interests

 Authors declare that they have no competing interests.

## Authors’ contributions

 LN, CJ, and MF designed the study. MF conducted the interviews and the data collection. LN, CJ, and MF interpreted the data. MF wrote a first draft and CJ and LN provided critical revision of the manuscript and supervision in the research. All authors read and approved the final manuscript.

## Funding

 This work was supported by public grants from the French National Cancer Institute [INCa, n° PREV19-017].

## Supplementary files


Supplementary file 1. Experts and Interviews Characteristics.

Supplementary file 2. Some Stakeholders Statements Retrieved From the Netnography, Classified From High to Low Influence Stakeholders.
