# Supplementary material for: Legitimacy of Front-of-Pack Nutrition Labels: Controversy Over the Deployment of the Nutri-Score in Italy
Source: Int J Health Policy Manag. 2022 Feb 20;11(11):2574–87. doi: 10.34172/ijhpm.2022.6127 (PMC9818094; doi:10.34172/ijhpm.2022.6127)
Supplement: Supplementary file 1 — Experts and Interviews Characteristics. [file ijhpm-11-2574-s001.pdf]

Article title: Legitimacy of Front-of-Pack Nutrition Labels: Controversy Over the Deployment of the Nutri-Score in Italy

Journal name: International Journal of Health Policy and Management (IJHPM)

Authors' information: Morgane Fialon[1]*, Lydiane Nabec[2], Chantal Julia[1,3]

[1]Nutritional Epidemiology Research Team (EREN), Sorbonne Paris Nord University/INSERM U1153/INRAE U1125/CNAM, Epidemiology and Statistics Research Center, University of Paris (CRESS), Paris, France.

[2]Centre de Recherche Réseaux, Innovation, Territoire et Mondialisation (RITM), Université ParisSaclay, Paris, France.

[3]Public Health Department, Avicenne Hospital, Assistance Publique des Hôpitaux de Paris (AP-HP), Paris, France.

(*Corresponding author: m.fialon@eren.smbh.univ-paris13.fr)

Supplementary file 1. Experts and Interviews Characteristics

**EXPERT**			**INTERVIEW**	
NAME IN THE TEXT	NATIONALITY	ORGANIZATION TYPE	DATE	LANGUAGE USED
Expert 1	Italian	Italian online newspaper	2020/03/10	French
Expert 2	Italian	Italian consumer association	2020/03/20	French
Expert 3	Italian	Italian public health institute	2020/04/28	Italian
Expert 4	Italian	Italian public health institute	2020/05/28	English
Expert 5	French	French research structure	2020/06/10	French
Expert 6	French	French directorate of health	2020/06/19	French
Expert 7	Italian *(working in France)*	French research structure	2020/08/24	French
Expert 8	Italian	Italian online newspaper	2021/09/17	English
