# Supplementary material for: Legitimacy of Front-of-Pack Nutrition Labels: Controversy Over the Deployment of the Nutri-Score in Italy
Source: Int J Health Policy Manag. 2022 Feb 20;11(11):2574–87. doi: 10.34172/ijhpm.2022.6127 (PMC9818094; doi:10.34172/ijhpm.2022.6127)
Supplement: Supplementary file 2 — Some Stakeholders Statements Retrieved From the Netnography, Classified From High to Low Influence Stakeholders. [file ijhpm-11-2574-s002.pdf]

Article title: Legitimacy of Front-of-Pack Nutrition Labels: Controversy Over the Deployment of the Nutri-Score in Italy

Journal name: International Journal of Health Policy and Management (IJHPM)

Authors' information: Morgane Fialon[1]*, Lydiane Nabec[2], Chantal Julia[1,3]

[1]Nutritional Epidemiology Research Team (EREN), Sorbonne Paris Nord University/INSERM U1153/INRAE U1125/CNAM, Epidemiology and Statistics Research Center, University of Paris (CRESS), Paris, France.

[2]Centre de Recherche Réseaux, Innovation, Territoire et Mondialisation (RITM), Université ParisSaclay, Paris, France.

[3]Public Health Department, Avicenne Hospital, Assistance Publique des Hôpitaux de Paris (AP-HP), Paris, France.

(*Corresponding author: m.fialon@eren.smbh.univ-paris13.fr)

Supplementary file 2. Some Stakeholders Statements Retrieved From the Netnography, Classified From High to Low Influence Stakeholders

MAIN CATEGORY OF ACTOR	ACTOR	CITATION	SOURCE
**Professional unions**<br>*(High influence)*	**Coldiretti**	*"The traffic light labeling system [Nutri-Score] is misleading, discriminatory and incomplete and - underlines Prandini - paradoxically ends up excluding from the diet healthy and natural foods that have been on the tables for centuries in favor of artificial products of which in some cases the recipe is not even known. The risk is - Prandini points out - to promote junk food with sweeteners instead of sugar and to reject the elixir of life such as* ***extra virgin olive oil,*** *considered the symbol of the* ***Mediterranean diet,*** *but also specialties such as* ***Grana Padano, Parmigiano Reggiano and Parma ham****, whose simple recipes cannot be changed."*	Today[1]
		Immediate reactions from Italy, which already at the end of 2018 had managed to foil the attempt of the UN [United Nations] to promote alarmist labels that would have affected the typical ***Made in Italy****. "Timely and wrong decision", comments Coldiretti, "they are misleading labels that, with simplification, risk to support dangerous models for health and for the* ***Made in Italy*** *quality production system and to promote junk food".*	La Repubblica[2]

	**Federalimentare**	*These systems, be they the English "traffic lights", the French "Nutriscore", or the black octagons adopted by Chile and Peru, do not induce consumers to make healthier choices, since they put the emphasis on individual foods rather than on the diet as a whole. In fact, leading scientists and nutritionists agree that a good diet is achieved through a varied and balanced diet, with appropriate intakes of all nutrients. Color-coded labeling systems, on the other hand, tend to discourage consumers from buying all products high in salt, saturated fat or sugar, branding them as unhealthy. These products include all the most* ***typical foods*** *of the* ***Italian tradition*** *and of the* ***Mediterranean diet****, despite their unquestionable quality". [...] "All the main health indicators rank Italy among the healthiest countries in the world, starting with longevity. - said Ivano Vacondio, president of Federalimentare - One of the main reasons for this state of grace is our diet based on the* ***Mediterranean diet****, whose healthiness is certified by 50 years of studies. On the contrary, an algorithm like the one on which the Nutriscore is based, cannot boast scientific bases consolidated over the years that are so solid as to suggest to populations to drastically change their eating habits" - concluded the president.*	Today[1]
		Federalimentare, Ivano Vacondio. "Our main fear - he said - is that behind the nutritional aspects there are actually other marketing and market aspects. Today, Italy boasts the most popular and sought-after cuisine in the world. A supremacy that we have achieved to the detriment of the French and I fear that the reconquest of this leadership, even penalizing the ***Made in Italy****, is the basis of these initiatives. The French traffic light label would be for us a disaster worse than the US duties".*	Il Sole 24 ore[3]
	**Cia-Agricoltori Italiani**	*The Nutri-Score label contributes to confuse consumers and wrongly penalizes the* ***Made in Italy****. Cia Agricoltori Italiani comments on the decision of the multinational Nestlé to use the Nutri-score by 2019 on all its products sold in European countries.*<br>*"The color-coded scheme -Cia recalls- provides a simplistic and distorted judgment on the single food, erasing in one fell swoop the assumption universally recognized by the scientific world that there are no "good" and "bad" foods, but rather correct or not correct diets depending on the way in which foods are integrated with each other on a daily basis. This type of labeling has, on its side, the extreme communicative simplicity (green is good, red is bad). However, it puts at risk many quality agro-food products, first and foremost Italian ones, bringing more harm than good. [...] Above all, by assigning the green light to a light beverage, with less sugar but rich in sweeteners, preservatives and flavorings, while giving the red light to products such as whole milk or cheese, oils, smoked fish, dried fruit and all the major* ***PDO (Protected Designation of Origin)*** *and* ***PGI (Protected Geographical Indications)*** *products such as* ***Grana****,* ***Parmigiano****,* ***hams and salami****, due to their natural fat content [...] As Italy, we relaunch, rather, the proposal of a "battery" system, based not on colors but on the indication of nutrients taken"*	Agricultura.it[4]
**Government**<br>*(High influence)*	**Ministry of Agriculture (MiPAAF)**	*The Minister of Agriculture Teresa Bellanova a few days ago in Brussels reiterated the negative opinion towards the Nutri-Score. "We do not like the red labels. They do not give correct nutritional information to citizens and penalize in a discriminating way many products of the* ***Mediterranean diet*** *or great* ***Italian PDO*** *and other countries. How can we say that we are promoting the quality of the territories with* ***PDO*** *and* ***PGI****, if then on the packaging together with the European quality mark there is a red label of rejection? It is not acceptable". The speech concluded by recalling that Italy is completing the experimentation of an alternative battery system. It is a position in line with the thesis of Coldiretti, Federalimentare and also of the Minister of Health Roberto Speranza who a few days ago issued similar statements.*	Il Fatto Alimentare[5]
		"We said it in Europe on Monday and I want to repeat it again here today: we cannot be victims of national algorithms. How can you think that next to the European ***PDO*** *mark on Grana Padano then you can find the nutriscore that puts the orange or red traffic light? It is unacceptable. - thunders the Minister of Agriculture, Teresa Bellanova, who spoke at the meeting in videoconference - We want full protection and we are working for an alternative such as the battery system that considers the daily needs and does not demonize individual ingredients. If we are the second country in the world for longevity after Japan, it is also the result of our diet. So I think we should be listened to on what to put on the label. For this reason, a true alliance with consumers is also necessary in Italy. Today, reiterating a concept that is crucial for us, it has been said that those who buy want to know what they are putting on their plates and, even before that, in their shopping carts. They want to make informed choices. They claim the right to be informed. And to be able to*	Il NordEst Quotidiano[6]

		orient themselves in the best way possible. It is important to work in this direction. Large-scale distribution plays a central role for both producers and consumers and we need to bring all parties into dialogue with transparency. We need spaces dedicated to our geographic brands, we need an enhancement in terms of product presentation."	
**Political Parties**<br><br>*(High influence)*	**Lega**	*"Nutri-Score, a score for foods. A project of EU [European Union] geniuses to say that the* ***Mediterranean diet*** *is bad for you. Let no one dare to outlaw the products of our sea and our land."*	Twitter (@matteosalvinimi)[7]
		On the subject Salvini had declared: "There is another negotiation kept hidden in Brussels, the one called Nutriscore. A stamp on foods with a red, yellow or green traffic light to say those that are good or bad for you. Foods like ***olive oil*** *or* ***prosciutto San Daniele*** *or* ***Pecorino Romano*** *would have a red light. It's a secret paper. It's a crazy boondoggle."*	ilGiornale.it[8]
	**Fratelli d'Italia**	*"Do you know what the #NutriScore is? A labeling program by a French government agency that favors French products over Italian ones."; "While the French are inventing the #Nutriscore to attack Italian products, the best diets ranking 2020 certifies that the #****Mediterranean Diet*** *is the best in the world. Let's defend our products, our* ***traditions*** *and our lifestyle."*	Twitter (@GiorgiaMeloni)[9]
		"The government is committed in the European Union to oppose the hypothesis of adoption of the 'Nutri-score' as a uniform labeling system likely to convey distorting nutritional messages and potentially penalizing and harmful to the national economy. Italy is universally recognized as a nation that exports quality of life and food. Initiatives are needed to preserve and protect the Italian food sector and the excellence of ***Made in Italy*** *from possible distorting effects on competition and fair international economic competition."*	Fratelli-Italia.it[10]
	**Other political parties**	*The "no" of Italian politics*<br>*The battle against the French classification system - not binding, but already adopted in France, Germany, Belgium, Spain and Holland - continues. On the one hand there is politics, with the approval on February 12 of a number of motions against the nutri-score. The front is united and the parties are fighting for a common goal.*<br>*The paper gathers speaking of left-wing parties: Antonio Tasso (MISTO-MAIE); Frederci Fornaro (LEU); Maria Chiar Gadda (IV); (PD) and right-wing parties mentioning that "Stronger positions are taken by right-wing parties" with: Luca de Carlo (FDI); Paolo Russo (FI); Lorenzo Viviani (LEGA); Luciano Cillis (M5S)*	Il Foro[11]
**Italian traditional food consortiums**<br><br>*(High influence)*	**Consorzio Parmigiano Reggiano**	*"No battery or traffic light labels on* ***Parmigiano Reggiano*** *and* ***PDO*** *and* ***PGI*** *products as proposed by the government and Federalimentare". The words of Nicola Bertinelli, president of the Parmigiano consortium, are clear when he says that the cheese "does not need a logo on the label to reassure the consumer", and justifies his position by highlighting the quality and the "recognition by nutritionists as a cheese with very high nutritional values, rich in proteins, vitamins and mineral salts".*	Il Fatto Alimentare[12]
	**Consorzio Grana Padano**	*"We are very pleased to hear these words of Minister Bellanova [see above] because they are perfectly in line with what we have always asked of the institutions, both Italian and EU [European Union] - commented the director general of the Consortium for the Protection of* ***Grana Padano PDO****, Stefano Berni -. The proposal put forward by the Consortium is clear and to the point: we are asking for a measure that guarantees the consumer to make an informed choice about what he or she is buying or consuming: on the shelves of large-scale retailers, products with a Denomination of Origin should always be clearly and unequivocally separated from their respective 'similar' products, just as restaurant menus should clearly indicate whether Denomination of Origin products or different homologues are being used."*	Il NordEst Quotidiano[6]
**Government**<br><br>*(Medium-high influence)*	**Ministry of Economic Development (MISE)**	*As Coldiretti, do you have any initiatives in store in the immediate future?*<br>*"I have met with Matteo Salvini, Antonio Tajani (Forza Italia, MEP), Paolo de Castro (Partito Democratito, MEP) and I have also spoken with the Ministers of Agriculture Teresa Bellanova and Economic Development Stefano Patuanelli so that a battery system is introduced, that is, one that takes into account the food diet and does not focus on the individual product."*	ilGiornale.it[13]

	**Ministry of Health**	*Even the Minister of Health Roberto Speranza says no to the "nutriscore", the system of the so-called "traffic light label" created to signal to consumers foods with a high presence of fat or salt. Speranza clarified his position speaking at a conference of Coldiretti and saying he was concerned that the Nutriscore could penalize the "**Made in Italy**" at the table. "A traffic light model in which canola oil is rated better than **olive oil** will never be accepted. We defend the **Made in Italy**, Italian companies," said the minister. He then added that "our message is deeper: we tell Europe that we want to be at the forefront on the quality of nutrition, not only for an Italian interest, but European."*<br>*"We have notified to the Commission the Italian proposal for nutritional labelling of nutritional products, nutrinform battery, we like this idea, we consider this proposal sustainable compared to the French nutri-score. Nutri-score fails to restore the quality of our **Mediterranean diet**, here it is not only about defending Italian products, our food production, here it is also about reflecting a lifestyle and eating habits that the world envies us, for which we are famous in the world. Therefore, in default, in the absence of proven scientific reasons, we cannot accept the imposition of a labeling mechanism that could disadvantage our products".*	Corriere della serra[14]
**Parliament**<br><br>*(Medium-high influence)*	**Chamber of Deputies**	*The issue of "nutritional labels" continues to be discussed while waiting for the final decision of the European Commission. Here are some positions. Cia Agricoltori Italiani expresses satisfaction for the unanimous vote in the Chamber with which the deputies of the Republic have approved, in recent days, a motion that commits the Government to oppose the French nutritional labeling system Nutri-score. Cia reminds that the Italian proposal for a nutritional labeling system for food products, which aims to be a valid alternative to the Nutri-score system, has just been notified (27 January 2020 ndr) to the European Commission.*	Informa Cibo[15]
	**Senate of the Republic**	*Commits the Government:*<br>*1) to take vigorous action at European level through the activation of all useful instruments to oppose the hypothesis of the adoption of the "Nutri-score", as a uniform labelling system likely to convey distorting nutritional messages and potentially penalizing and harmful to the national economy;*<br>*2) to preserve and protect the Italian food sector and the excellence of **Made in Italy** from possible distorting effects on competition and fair international economic competition of European and internal policies within the common market developed on the basis of initiatives by individual governments of other Member States and characterized by not inconsiderable elements of hostility and aggressiveness, as in the case of the Nutri-score;*<br>*3) to propose, alternatively, at European level, a system of foodstuff labelling suitable for relaunching the fundamental need to spread the **Mediterranean diet**, recognized worldwide by UNESCO as an intangible transnational asset, and to integrate the Italian proposal for a battery label for foods with a visual reference to this diet;*<br>*4) to promote, both at European and international and global level, the recognition of the importance of the specific **PDO** and **PGI** foodstuffs and their profound cultural as well as food value, promoting specific labelling that enhances these products and excludes or exempts them from the obligation to adopt systems that do not distinguish them from any other foodstuff lacking the peculiar and unrepeatable characteristics that mark them.*	Senate website[16]
**National Institutes**<br><br>*(Medium-high influence)*	**ISS**	*At the end of November - as revealed in recent days by some news agencies and confirmed by Mipaaf - the experimentation was concluded, which demonstrated how "the voluntary battery system [NutrInform Battery] facilitates the consumer's understanding of the contribution or importance of the food for the purposes of energy and nutrient intake". The path that is leading to the adoption of the new label began in November 2017 with the establishment of a working group between representatives of ministries and associations of the agri-food chain, which developed an initial proposal. Following two phases of experimentation, a memorandum of understanding was signed in April 2019 between the ministries involved, the Istituto Superiore di Sanità (ISS) and CREA.*	La Repubblica[2]
	**CREA**	*For the realization of this project [NutrInform Battery], led by four Ministries (Health, Economic Development, Agriculture and Foreign Affairs), nutritional experts from the National Institute of Health (ISS) and the Council for Economic and Agriculture Research (CREA), as well as representatives of trade associations of the agri-food chain and consumers, took the field.*	NutrInformBattery.it[17]

**Consumer associations** *(Low influence)*	**AltroConsumo**	*However, consumer campaigners claim the Italian alternative to Nutri-Score is "counter-intuitive" and "confusing". Italian consumer group Altroconsumo said it favours Nutri-Score. "A nutritional label should be based on uniform reference amounts (such as per 100g/100ml) and should use a color-coding to help consumers to compare the nutritional value of foods across a range of products. This is the Nutri-Score", a spokesperson told FoodNavigator.*	FoodNavigator[18]
	**Condacons**	*Net, finally, the rejection of Codacons, "it seems a joke where some products are bad regardless, without taking into account the amount consumed."*	La Repubblica[2]
**Scientists** *(Low influence)*	**Silvio Garattini, Walter Ricciardi, Mario Serafini, Paolo Vineis and Elio Riboli**	*The bitter observation is that in Italy, when important food issues have to be dealt with, the opinion is that of lobbies and politicians, not that of nutritionists or scientific societies. Experts are not taken into consideration. Those who know the difficulty of people struggling with the list of ingredients or with the numbers of the nutritional table appreciate the traffic light. Even authoritative scientists such as Silvio Garattini, Walter Ricciardi, Mario Serafini, Paolo Vineis and Elio Riboli have signed an appeal in favor of the Nutri-Score, but this matters little to politicians. In Italy, the opinion of Coldiretti is worth as much as a decree, and is proposed by ministers who probably have not consulted the relevant bodies such as CREA, ISS or CNSA [Ministry of Health]. Fortunately, the Italian no has little value in Europe. The traffic light label is a voluntary choice of individual companies and no one can hinder it since it is foreseen by the rules. In Italy there are supermarket chains that have been following the Nutri-Score for years, and some have even thought of adopting it for branded products.*	Il Fatto Alimentare[5]
	**Walter Ricciardi**	*Nevertheless, La Stampa also highlights the opinion of Walter Ricciardi, a transalpine doctor who defends the French labeling system: "Some people say that Nutri-Score penalizes **Parmigiano Reggiano**, **mozzarella** or **olive oil**. This is nonsense, because the C value only means 'do not exaggerate' and D 'eat once a week'. France does not try to favor its cheese. In this country, this mechanism has already led to a small reversal of the trend among consumers. Such a system could therefore also stimulate Italian producers to be more careful in the ingredients they use."*	La Stampa[19]
	**Walter Ricciardi**	*Nutriscore: doctor Walter Ricciardi: "No food in itself is dangerous, even the fattiest and most sugary foods can be consumed in a balanced diet. This product was opposed by French cheesemakers in the same way that Italian producers opposed it."*	LA7 (TV)[20]
	**Silvio Garattini, Walter Ricciardi, Mario Serafini, Paolo Vineis and Elio Riboli**	*The nutritional information initiative "Nutri-Score", developed by independent university researchers from the University of Paris and INSERM, has been the object of various attacks and distancing in Italy in recent days, misunderstanding it as a tool through which Europe would like to penalize Italian products and the **Mediterranean diet**.*<br>*Again, **olive oil**, in the Nutri-Score, has the best score for added fats (C), and not, as has been incorrectly said, the "red light". And this in absolute coherence with the recommendations for public health that, in Italy as elsewhere, encourage consumers to favor it over other vegetable oils and especially over animal fats.*	Scienza in Rete[21]

References of Supplementary file 2:

1. Nutri-Score, l'etichetta "semaforo" per alimenti: cos'è e perché potrebbe essere ingannevole. Today. https://www.today.it/economia/etichetta-nutri-score.html. Accessed September 9, 2021.
2. L'Italia verso l'etichetta a batteria. Un nuovo logo dirà quanto ci stiamo "ricaricando." la Repubblica. https://www.repubblica.it/economia/diritti-e-consumi/diritti-consumatori/2020/01/29/news/l_italia_verso_l_etichetta_a_batteria_un_nuovo_logo_con_i_nutrienti_dei_cibi-246605082/. Published January 30, 2020. Accessed September 16, 2021.

3. La Francia sperimenta nelle mense la contestata etichetta a semaforo - Il Sole 24 ORE. https://www.ilsole24ore.com/art/la-francia-sperimenta-mense-contestata-etichetta-semaforo-AC7MW21?refresh_ce=1. Accessed September 20, 2021.
4. Agricultura.it. Nutri-score Nestlé. Cia, da etichetta a semaforo più danni che benefici. *Agricultura.it*. June 2019. https://www.agricultura.it/2019/06/27/nutri-score-nestle-cia-da-etichetta-a-semaforo-piu-danni-che-benefici/. Accessed September 14, 2021.
5. Pira RL. La guerra delle etichette: Bellanova e Speranza contrari al Nutri-Score! Ma non servono autorizzazioni e il semaforo avanza in Europa. *Il Fatto Alimentare*. https://ilfattoalimentare.it/nutri-score-etichetta-semaforo-bellanova.html. Accessed September 14, 2021.
6. Redazione. Consorzio Grana Padano: nel 2019 prodotte 5.200.000 forme (+5,06%). *Il NordEst Quotidiano*. December 2019. https://www.ilnordestquotidiano.it/2019/12/18/consorzio-grana-padano-nel-2019-prodotte-5-200-000-forme-506/. Accessed September 24, 2021.
7. nutriscore OR nutri-score (from:matteosalvinimi) until:2020-02-17 since:2019-11-01 - Recherche sur Twitter / Twitter. Twitter. https://twitter.com/search?q=nutriscore%20OR%20nutri-score%20(from%3Amatteosalvinimi)%20until%3A2020-02-17%20since%3A2019-11-01. Accessed September 14, 2021.
8. Salvini: "Nella Nutella nocciole turche. Preferisco mangiare italiano." ilGiornale.it. https://www.ilgiornale.it/news/politica/salvini-nella-nutella-nocciole-turche-preferisco-mangiare-1795004.html. Published December 5, 2019. Accessed September 14, 2021.
9. nutri score (from:GiorgiaMeloni) until:2020-02-21 since:2019-06-26 - Recherche sur Twitter / Twitter. Twitter. https://twitter.com/search?q=nutri%20score%20(from%3AGiorgiaMeloni)%20until%3A2020-02-21%20since%3A2019-06-26. Accessed September 14, 2021.
10. Made in Italy, FdI: Governo si impegni per contrastare nutriscore. *Fratelli d'Italia*. February 2020. https://www.fratelli-italia.it/2020/02/12/made-in-italy-fdi-governo-si-impegni-per-contrastare-nutriscore/. Accessed September 14, 2021.
11. Nutri-score: l'etichetta che minaccia il made in Italy. *IL FORO*. February 2020. https://www.ilforo.eu/2020/02/16/nutri-score-letichetta-che-minaccia-il-made-in-italy/. Accessed September 20, 2021.
12. Pira RL. Parmigiano Reggiano dice no all'etichetta a batteria proposta dal Governo e anche al semaforo francese. *Il Fatto Alimentare*. https://ilfattoalimentare.it/etichetta-semaforo-batteria-parmigiano-reggiano.html. Accessed September 20, 2021.
13. Il bollino rosso ai nostri cibi inganno delle multinazionali. ilGiornale.it. https://www.ilgiornale.it/news/politica/bollino-rosso-ai-nostri-cibi-inganno-delle-multinazionali-1795029.html. Published December 6, 2019. Accessed September 14, 2021.
14. Il ministro Speranza: «No all'etichetta a semaforo per il cibo». Corriere della Sera. https://www.corriere.it/politica/19_dicembre_19/ministro-speranza-nutriscore-dico-no-all-etichetta-semaforo-il-cibo-d9e7bf36-226c-11ea-8e32-6247f341a5cc.shtml. Published December 19, 2019. Accessed September 16, 2021.
15. Cia e Coldiretti: bene il voto della Camera contro il Nutriscore francese. InformaCibo. https://www.informacibo.it/cia-e-coldiretti-bene-il-voto-della-camera-contro-il-nutriscore-francese/. Published February 19, 2020. Accessed September 16, 2021.
16. Legislatura 18ª - Aula - Resoconto stenografico della seduta n. 185 del 29/01/2020. http://www.senato.it/japp/bgt/showdoc/frame.jsp?tipodoc=Resaula&leg=18&id=1141906&part=doc_dc-allegatob_ab. Accessed September 16, 2021.
17. NutrInform Battery. Nutrinform Battery. https://www.nutrinformbattery.it/. Published 2020. Accessed December 14, 2020.
18. foodnavigator.com. Does Nutri-Score discriminate against traditional foods? foodnavigator.com. https://www.foodnavigator.com/Article/2020/12/11/Does-Nutri-Score-discriminate-against-traditional-foods. Published December 11, 2020. Accessed December 15, 2020.
19. ufficiostampa. » NESTLE', CODACONS: SEMAFORO IN ETICHETTA FUORVIANTE E INGANNEVOLE PER I CONSUMATORI. https://codacons.it/nestle-codacons-semaforo-in-etichetta-fuorviante-e-ingannevole-per-i-consumatori/. Accessed September 9, 2021.
20. *Nutriscore, il medico Walter Ricciardi: "Mi baso sull'evidenza scientifica, ci sono studi che dimostrano che questa etichetta alimentare è uno strumento efficace."* https://www.la7.it/laria-che-tira/video/nutriscore-il-medico-walter-ricciardi-mi-baso-sullevidenza-scientifica-ci-sono-studi-che-dimostrano-31-01-2020-305138. Accessed September 20, 2021.
21. Nutri-Score: perché non dobbiamo averne paura. Scienza in rete. https://www.scienzainrete.it/articolo/nutri-score-perch%C3%A9-non-dobbiamo-averne-paura/2019-12-13. Published December 13, 2019. Accessed September 20, 2021.
